# Statistical Analysis and Tokenization of Epitopes to Construct Artificial Neoepitope Libraries

**DOI:** 10.1021/acssynbio.3c00201

**Published:** 2023-09-13

**Authors:** Elena Lopez-Martinez, Aitor Manteca, Noelia Ferruz, Aitziber L. Cortajarena

**Affiliations:** † Centre for Cooperative Research in Biomaterials (CIC biomaGUNE), Basque Research and Technology Alliance (BRTA), Paseo de Miramón 194, Donostia-San Sebastián, 20014 Spain; ‡ Molecular Biology Institute of Barcelona (IBMB-CSIC), Barcelona Science Park, Baldiri Reixac, 15-21, 08028, Barcelona, Spain; § IKERBASQUE, Basque Foundation for Science, Plaza Euskadi 5, 48009 Bilbao, Spain

**Keywords:** epitope analysis, library design, tokenization, natural language processing, byte pair encoding

## Abstract

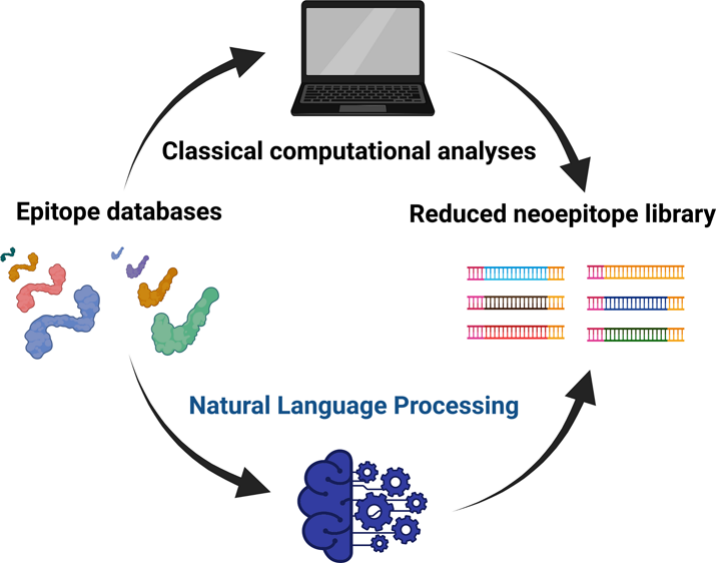

Epitopes are specific regions on an antigen’s surface that the immune system recognizes. Epitopes are usually protein regions on foreign immune-stimulating entities such as viruses and bacteria, and in some cases, endogenous proteins may act as antigens. Identifying epitopes is crucial for accelerating the development of vaccines and immunotherapies. However, mapping epitopes in pathogen proteomes is challenging using conventional methods. Screening artificial neoepitope libraries against antibodies can overcome this issue. Here, we applied conventional sequence analysis and methods inspired in natural language processing to reveal specific sequence patterns in the linear epitopes deposited in the Immune Epitope Database (www.iedb.org) that can serve as building blocks for the design of universal epitope libraries. Our results reveal that amino acid frequency in annotated linear epitopes differs from that in the human proteome. Aromatic residues are overrepresented, while the presence of cysteines is practically null in epitopes. Byte pair encoding tokenization shows high frequencies of tryptophan in tokens of 5, 6, and 7 amino acids, corroborating the findings of the conventional sequence analysis. These results can be applied to reduce the diversity of linear epitope libraries by orders of magnitude.

Peptidic epitopes are mainly small protein regions from microorganisms involved in noncovalent interactions with immune cells, such as T lymphocytes and antibodies. Epitopes can be classified into two groups based on their conformation and their interaction with the recognition site within the antibody, i.e., the paratope. Linear or sequential epitopes are recognized by the antibody because of their specific amino acid sequence. In this case, only the primary structure of the peptide is recognized by the antibody. In contrast, conformational epitopes require several discontinuous segments of the protein to play a role in the recognition. Detection is based on the secondary and tertiary structure of both the antibody and the antigenic protein. Thus, discovering epitopes in antigenic proteins is not always straightforward. These proteins may contain more than one linear epitope for different antibodies.^1,2^ Moreover, structural epitopes can be challenging to determine in scenarios without structural data, such as at the onset of a viral outbreak.

The prediction of antibody–antigen binding (Figure 1) is a central question in immunology. Finding epitopes in protein sequences can accelerate the development of vaccines^3^ and immunotherapies.^4^ Additionally, these sequences can be used to design in vitro diagnostic tests to assess the exposure to pathogens in humans, livestock, or vector animals and serve as a first line of defense to prevent and monitor future epidemics. All of these issues highlight the importance of having rapid techniques to define and characterize epitopes. Traditional methods to detect these sequences are based on experimental procedures such as cocrystallization,^5^ cryogenic electron microscopy (cryo-EM),^6^ phage display^7^ or array-based oligopeptide scanning.^8^ However, these protocols are costly and time-consuming, limiting the rapid response required for the development of diagnostic tools, vaccines, and immunotherapies. To address this issue, one alternative is to create a universal randomized epitope library that can be rapidly screened against any target antibody.

**Figure 1:**
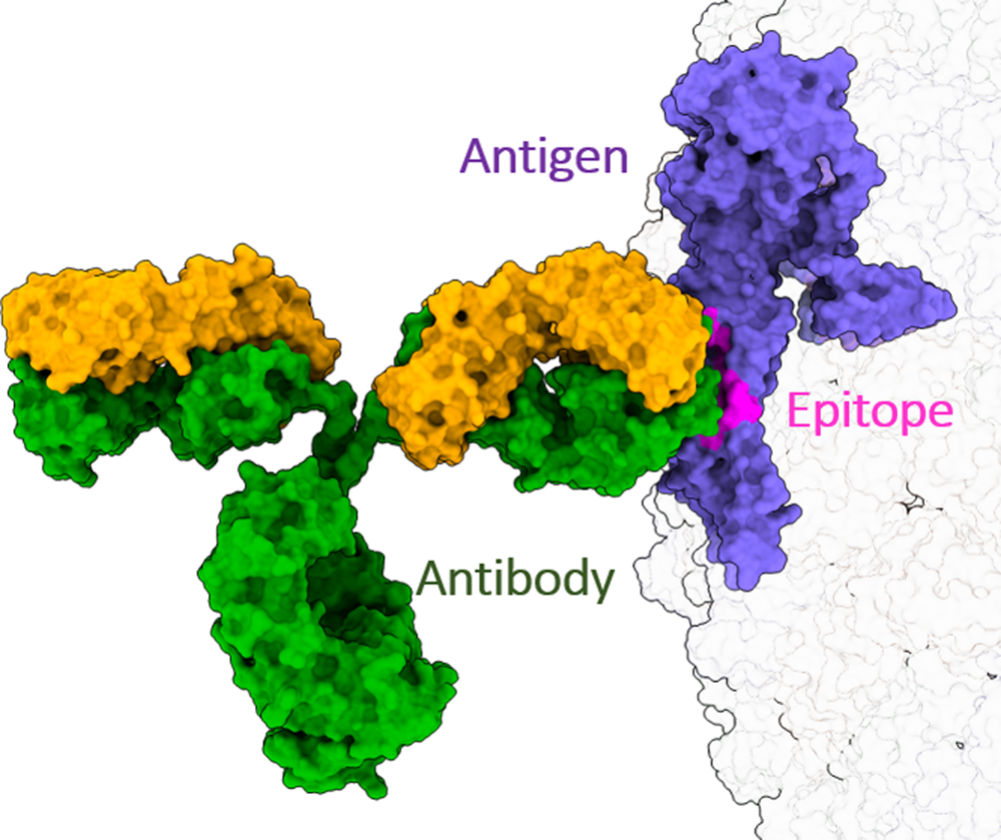
Structural scheme of antibody–antigen binding. The figure shows the antibody (heavy chains in green and light chains in yellow)–antigen (in purple) complex between the Zika virus E protein and the mouse monoclonal immunoglobulin G molecule (IgG). The 18-mer amino acid epitope within the E protein is highlighted in pink (montage made with PDBs: 1IGT and 5IRE).

Deep learning (DL) algorithms have shown great potential to tackle complex biological problems, including directed evolution of proteins,^9,10^ eukaryotic gene expression regulation,^11^ and modeling nucleic acid aptamers.^12^ Among all fields using DL, natural language processing (NLP) has demonstrated significant advancements in the last years, in particular, in language understanding and generation. NLP techniques are being applied in biological problems^13^ or design novel functional proteins.^14^ Here, we have applied classical computational analyses and an NLP-inspired tokenization algorithm to identify sequence patterns in linear epitopes deposited in the Immune Epitope Database (IEDB).^15^ The results can be used as building blocks for the design of neoepitope libraries.

## Results and Discussion

### Global Amino Acid Propensity in Epitopes

**Figure 2:**
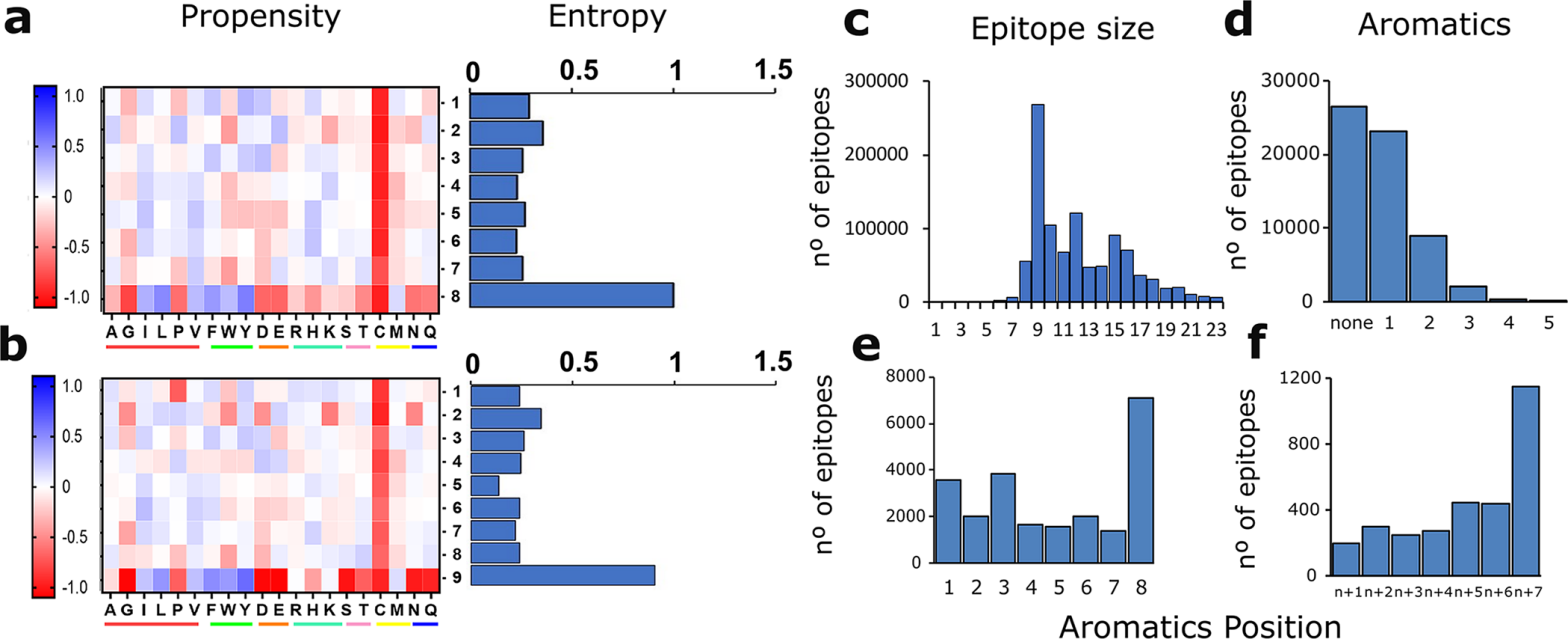
Computational analysis of the IEDB sequences. (a,b) Amino acid global propensity and entropy for 8-mer and 9-mer epitopes, respectively. The amino acids are underlined as follows: aliphatic in red, aromatic in green, acidic in orange, basic in blue, hydroxylic in pink, sulfur-containing in yellow, and amidic in dark blue. (c) Length of the analyzed epitopes. (d) Number of aromatic residues in 8-mer epitopes. (e,f) Position of aromatic residues in (e) 8-mer epitopes carrying a single aromatic residue and (f) 8-mer epitopes carrying 2 aromatic residues.

The predictability of antibody–antigen binding relies on the assumption that paratope-epitope interaction motifs are universally shared among the antibody–antigen structures. Some studies sought to establish statistical relationships in epitopes, but the number of sequences analyzed in these studies has been limited to a few hundreds or thousands, and they usually focus on conformational epitopes.^16,17^ These works analyzed structural data of antibody–antigen complexes and found a higher propensity of hydrophilic residues in epitopes and an enrichment of aromatic residues in paratopes. To investigate this phenomenon in a broader data set, we have performed a computational study considering the annotated epitopes in the IEDB. The IEDB contains experimental data on B cell and T cell epitopes, including entries related to infectious diseases, allergies, autoimmunity, and transplantation. Our statistical analysis revealed that epitopes share common features related to their overall amino acid frequency. Figure 2a and 2b depict the global propensity of each amino acid in epitopes with a length of 8 (*n* = 55,609) and 9 (*n* = 268,118) amino acids, respectively, compared with the human proteome (see Methods). Global propensity is an indicator used to measure the relative occurrence of amino acids for a given data set. These epitope lengths are two of the most represented in the entire data set (*n* = 716,529) (Figure 2c). The propensity for the rest of the epitopes is shown in Figure S1, and the overall global propensity variation is shown in Figure S2. The abundance of aromatic residues in epitopes is significant, especially in the last position of these short sequences, which is in contradiction with other reported aromatic residue propensities in conformational epitopes.^16,17^ This phenomenon could be explained by the potential pi-stacking interactions between the aromatic residues of the epitope and the antibody, which may play a role in the molecular recognition of this type of system.^19^ Pi-stacking has several implications in other biomolecular recognition processes.^20,21^ The long-range of interacting distances between aromatic residues could account for their underrepresentation in structural-based analyses, where contact residues are filtered based on a distance cutoff of <4.5 Å, which is suitable for capturing most molecular interactions in proteins, including hydrogen bonds and van der Waals forces. However, this cutoff excludes aromatic interactions, such as π-stacking, which occur at distances ranging from 4.5 to 7.5 Å.^18^ Another relevant phenomenon observed is the low propensity of cysteines in all of the epitope positions. Cysteines contain a sulfur atom that can form disulfide bonds with other cysteines. Disulfide bonds are strong, covalent-like bonds, with a typical bond dissociation energy of 60 kcal/mol.^22^ Antibody–antigen recognition is a high-affinity interaction yet reversible, and hence, cysteines are probably not good candidates for such rescindable binding.

Additionally, we computed the frequency and relative positions of the aromatic residues in the epitopes. Figure 2d shows that 56.5% of the 8-mer epitopes contain at least one aromatic residue, corroborating the previous propensity data. Figures 2e and 2f depict the position of the aromatic residues in the epitope with 1 or 2 aromatic amino acids. It can be observed that the probability of finding an aromatic residue at the last position of the epitope is higher than that at any other position. Considering this phenomenon, the relative entropy of each position in the epitope sequence was also examined. Relative entropy gives a numerical value of the amino acid variation at each position by calculating the separation of the amino acid distribution at each position in epitopes from a position-independent reference state, the amino acid frequency in all proteins in the human proteome (see Methods). This analysis revealed that the last position of the epitope is significantly more entropic than the others and that this phenomenon is independent of the length of the epitope (right panels of Figure 2a and 2b and Figure S3) up to 12-mers. The results obtained from all the data sets indicate average relative entropy values ranging from 0.15 to 0.3 (Table S1). These values signify the high variability observed in epitopes, which can be interpreted as a fundamental characteristic of their biological role. In fact, a relative entropy below 0.3 has previously been used to define hypervariable positions that play a key role in protein–protein interactions.^23^

### NLP-Inspired Tokenization of Epitopes

**Figure 3:**
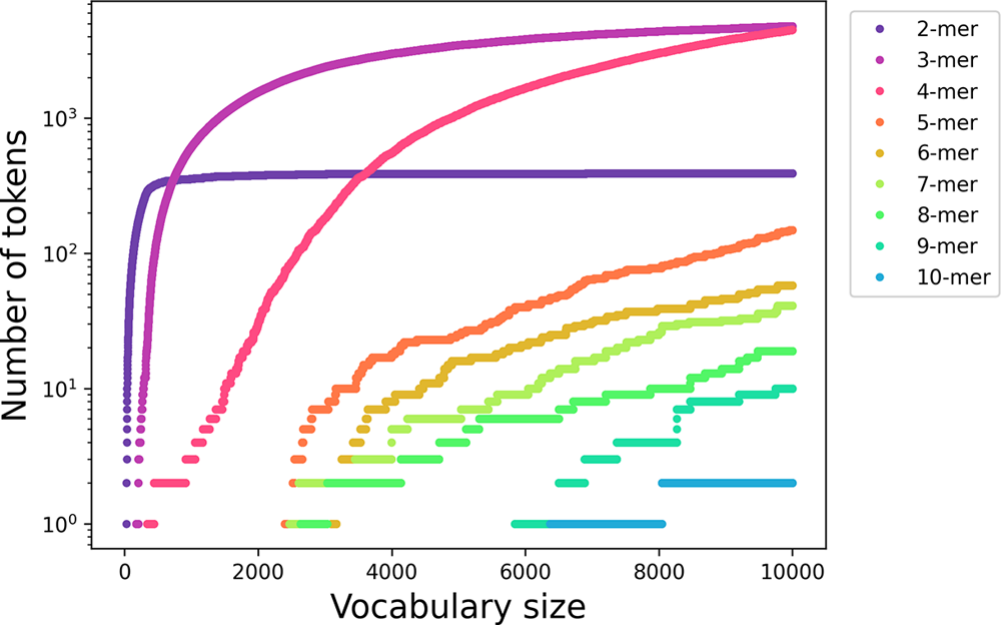
Number of tokens of a certain length found at each vocabulary size. BPE works sequentially, finding shorter tokens (2-mers, 3-mers, and 4-mers) first. These tokens tend to plateau at their limit; e.g., there are only 20^2^ possible 2-mers. Tokens of longer lengths only appear at larger vocabulary sizes; e.g., the first 10-mer appears with a vocabulary size of 6372.

**Figure 4:**
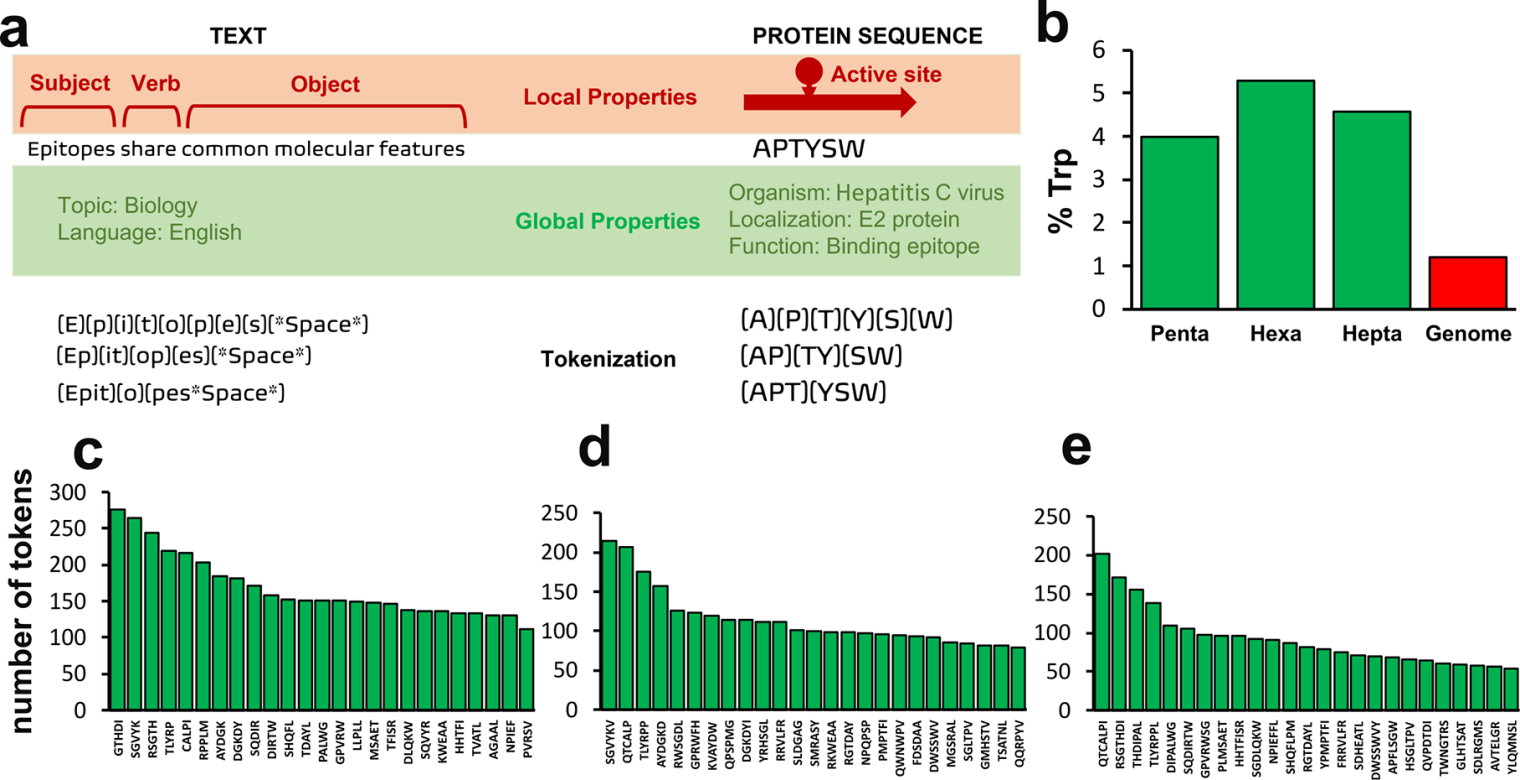
Tokenization of the IEDB with the BPE algorithm. (a) Comparison between NLP-inspired tokenization applied to human communication languages and protein languages. (b) Frequency of tryptophan residues in the 25 most represented tokens of 5, 6, and 7 amino acids and in the whole bacterial genome. (c–e) The 25 most represented tokens of 5, 6, and 7 residues, respectively. The *y* axis represents the number of tokens that appear in the given sequence.

The IEDB data set contains more than 10^6^ epitope entries and continues to grow daily. Analyses such as the search of protein motifs or repetitions require specific computational techniques. For instance, the EMBL’s European Bioinformatics Institute (EMBL-EBI) hosts the PRATT software for protein pattern analyses.^24^ This tool enables the identification of conserved patterns in sets of unaligned protein sequences. However, it is limited to analyzing only 100 protein sequences, highlighting the need for algorithms that enable high-throughput analyses. Other useful software, such as Pepsurf^25^ and MimoPro,^26^ are valuable tools for epitope mapping after previous selection of peptides using phage display technologies. However, these tools require structural data of the antibody to accurately compute the potential epitope sequences over the interaction surface of the paratope. In this context, DL algorithms have gained substantial importance in the field of bioinformatics^27^ over the past few years. In recent years, computer vision (CV) and NLP have witnessed remarkable advancements, culminating in the development of cutting-edge tools such as DALLE2 or ChatGPT. Interestingly, there exist significant parallels between human language and protein sequences.^28^ Not surprisingly, NLP techniques have been widely applied to the protein research realm, such as in homology detection or protein functional classifications.^28^ With the advent of highly performing language models, NLP is now accelerating the analysis and design of protein sequences,^29^ and has even allowed the prediction of protein structures with atomic accuracy.^30^ NLP methods also allow the tokenization (slicing an input in atomic units of information known as tokens) of strings (Figure 4a), facilitating the search for contiguous protein motifs and repetitions. In this work, we have used tokenization techniques to extract meaningful linear patterns from the epitope sequence data. The tokenization has been carried out using the byte pair encoding (BPE) algorithm.^31^ BPE is a data compression algorithm that replaces the most common consecutive pair of bytes of data with a byte that does not appear in the data set. Hence, it can also be used to find the most frequent bytes (or subwords) and has been widely adopted in NLP preprocessing steps due to its speed and performance. In the context of our epitope data set, BPE finds overrepresented tokens sequentially, starting from single amino acids and dipeptides and continuing with tokens of three, four, and five amino acids, respectively, until finding the longest token, which we set up to 10-mers in this study. Initially, we observed a significant proportion of tokens containing multiple repetitions of the same amino acid. Although poly-X patterns proteins have been associated with roles in disease,^32,33^ we have not found evidence in the literature suggesting their involvement in Ab-antigen recognition. For instance, our analysis of the IEDB database revealed that over 75% of 4-mers containing polyA-, poly-P, poly-S, and poly-G correspond to epitopes from *Trypanosoma cruzi* (Figure S4), which may indicate a bias toward well-studied pathogens with distinctive poly amino acid proteins, such as the mucin-like proteins from *T. cruzi*.^34^ As our goal is to define an epitope library that encompasses a wide range of pathogens, we have preprocessed the data set to filter out entries containing poly-X patterns of four or more identical amino acids. The final data set, including epitopes of all lengths, was tokenized with an increasing number of final tokens as a target (from 50 to 9950 tokens). Larger vocabulary sizes allow for the finding of longer tokens. Results revealed that tokens with lengths of 3 and 4 amino acids still exhibit a significant proportion of tripeptides with identical amino acids (e.g., “LLL”). To mitigate potential noise, we focus on tokens with larger lengths. Figure 3 summarizes the number of tokens of a certain length for each tokenized vocabulary size. BPE operates sequentially, and thus at small vocabulary sizes (<2000) 2-mers, 3-mers, and 4-mers are first found. The occurrence of these token sizes tends to plateau since there are only 20^2^, 20^3^, and 20^4^ combinations for each respective k-mer. Figure S5 illustrates the most common tokens of the 3-mers and 4-mers. At vocabulary sizes of 2000 and beyond, 5-mers and tokens of larger sizes begin to emerge. Tokens of those lengths showed an elevated frequency of tryptophan residues in the epitopes, which supports previous computational results. In particular, Figure 4b shows the frequency of tryptophan residues on the 25 most repeated tokens of 5, 6, and 7 residues versus the average frequency of tryptophan in 9 bacterial and archaeal genomes.^35^ The frequency of tryptophan is between 3 and 5 times higher in these tokens, suggesting that it may play an important role in the molecular recognition of linear epitopes. The 25 most repeated tokens of 5, 6, and 7 residues are shown in Figure 4c, 4d, and 4e, respectively. Interestingly, 59% of the 25 most represented tokens contained at least one aromatic residue. Figure S6 compares the amino acid frequencies for 5-mers, 6-mers, and 7-mers sets after tokenization with their natural frequencies. We observed additional trends that were not revealed in the statistical analyses. Specifically, we note the elevated frequencies of residues D, G, P, R, S, and Y in epitope tokens with lengths of 6 and 7, as well as the relatively low frequencies of residues E, I, K, and L when compared to the reference values.

This study provides useful insights into defining the sequences of randomized libraries that will help to find artificial epitopes. Since the construction of a universal library including all the possible epitopes appears unapproachable, optimizing the possible immunoresponsiveness of fewer sequences of a focused, smaller designed library can be an effective strategy. For instance, a completely randomized library of 6 residues possesses a diversity of 6.4 × 10^7^ variants, whereas fixing one of these positions to tryptophan would decrease this diversity by 20-fold. Furthermore, the tokenization of the data set also provides means to reduce the size of the epitope library for further studies. Table S2 summarizes the number of tokens for different vocabulary sizes and their respective coverage of the entire data set. For example, the 25 4-mers found at a vocabulary size of 2000 cover 1.4% of the entire data set. These tokens allow for further position fixing; e.g., creating a library with these 4-mers at all possible positions of 6-residue library would reduce its variability to 10^4^. Another possibility is the use of degenerated codons. Generating a library with 4 RVK codons, encoding charged hydrophilic residues (A, D, E, G, H, K, N, R, S, T) and 2 YWC codons, enriched in aromatic residues (F, H, L, Y) will reduce the diversity by 2 orders of magnitude. Moreover, considering the results of the tokenization, it is possible to additionally shorten the protein sequence space by locking one or two codons to a single amino acid, such as W or Y. These reductions can be applied in countless possible combinations to fine-tune the diversity of the library in a custom manner, fixing a restrictive relative entropy in positions where diversity is not needed.

## CONCLUSIONS AND OUTLOOK

In this study, we applied both classic statistical methods and NLP-inspired algorithms to identify universal patterns in linear epitopes deposited in the IEDB. Our results suggest that certain trends, such as the patterned presence of aromatic residues or the low frequency of cysteine residues, are common in linear epitopes. These computational analyses aim to reduce the size of the protein epitope libraries by minimizing the randomization of the residues or fixing certain positions to a single amino acid. This reduction in size will allow for a more efficient screening of the library using various techniques.

Furthermore, the identification of patterns in epitopes provides valuable insights for designing therapies and vaccines based on the antibody–antigen interaction, for example, using high-throughput data from epitope libraries to train ML algorithms to better predict epitopes involved in specific antibody–antigen interactions. Consequently, better strategies can be employed to neutralize pathogens and boost the humoral and immune response.^36,37^ In the diagnostics field, the COVID-19 pandemic has shown that the rapid development of reliable antibody/antigen detecting devices is paramount for the early detection and control of new infectious pathogens. The fast production of such devices can help in evaluating the exposure to pathogens and levels of immunization after treatments or vaccination. Moreover, the mass fabrication of these point-of-care diagnostic devices has several advantages in public-health control. All of these factors make it clear that fast, cheap, and easy-to-use antibody/antigen detection devices will gain importance exponentially in the following decades. The design of artificial neoepitopes can accelerate all these processes, allowing the development of tests for new pathogens or strains without the need of established structural data from pathogens’ proteins.

The advancements in machine learning, specifically NLP algorithms, have emerged as significant contributors in extracting meaningful information from expansive protein data sets. Notably, the BPE algorithm has demonstrated efficacy in routinely identifying prevalent linear patterns in vast data sets. Furthermore, the field continues to expand at an unprecedented pace, with recent developments including the use of generative models for constructing antibody libraries.^38^ Machine learning and NLP algorithms are proving to be pivotal in analyzing protein data sets, facilitating a deeper understanding of protein structure, function, and interactions.

Finally, understanding the molecular mechanisms that govern epitope recognition could pave the way for the development of artificial T cell-like complexes. The engineering of cells with the capacity to recognize specific pathogenic epitopes would significantly advance the field of artificial-cell therapies. In this context, previous research articles have reported an enrichment of aromatic residues in the paratope segment of the antibodies.^17,39−41^ These results, together with our findings on the elevated presence of aromatic residues also in the epitopes, could clarify the role of pi-stacking in immunomolecular recognition. We hypothesize that these aromatic residues in both the epitope and the antibody could act as an interdigitated molecular zipper playing a key role in the molecular recognition and allowing for quick and reversible complex binding.

## Methods

### Global Propensity and Relative Entropy Analyses

Statistical calculations were conducted downloading the nonpost-translation modified linear epitope sequences database from www.iedb.com. Amino acid propensity was determined with the standardized methods used elsewhere.^42^ Thus, global propensities (GP) for each amino acid at each position have been calculated as follows:
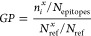
where *n*_*i*_^*x*^ is the number of epitope sequences that contain the amino acid *x* at position *i*, *N*_epitopes_ is the total number of epitope sequences, *N*_ref_^*x*^ is the total number of each amino acid *x* in all positions in the reference set, and *N*_ref_ is the total number of positions in the reference set. The reference set of the human proteome codon usage was obtained from ref (43). The amino acid frequencies *f*(*x*) for each amino acid *x* are A (0.07), C (0.023), D (0.047), E (0.071), F (0.036), G (0.066), H (0.026), I (0.043), K (0.057), L (0.1), M (0.021), N (0.036), P (0.063), Q (0.048), R (0.056), S (0.083), T (0.054), V (0.06), W (0.012), and Y (0.027). Logarithmic values of the global propensities were used in the heatmap plots to normalize the data. Relative entropy calculations were carried out with the regular protein engineering methodologies to calculate entropies used in other articles.^23^ Thus, relative entropy is calculated using the following equation:
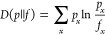
where *D* is the relative entropy and *p*_*x*_ is the proportion of sequences with amino acid *x* at position *i*. A single factor ANOVA analysis was performed to obtain the *p*-values for each X-mer data set. The null hypothesis states that the mean entropies at each position are equal, and the significance level used to reject the null hypothesis is α = 0.05.

### Tokenization

Byte Pair Encoding was used to tokenize the epitope data set. Before tokenizing, we removed all sequences with five or more contiguous amino acids of the same type (e.g: “AAAAA”). We used the Hugging Face library^44^ in a data set size is 716,529 epitopes and excluded the new line character (“\n”) from the process. We tokenized from vocabulary sizes ranging from 50 to 10000. The average tokenization runtime for a certain vocabulary size is 27.87 s on a standard workstation.

### Data Visualization

Graphs and figures were made using GraphPad Prism and Inkscape. Table of contents image has been designed using www.biorender.com.

## Supporting Information Available

Figure S1. Amino acid global propensity of epitopes. Figure S2. Overall global propensity variation in epitopes. Figure S3. Relative entropy of epitopes. Figure S4. Distribution of epitopes by source organism. Figure S5. Tokenization of epitopes with tokens of 4 and 5 residues. Figure S6. Amino acid frequency after tokenization. Table S1: Average relative entropies and p-values for the entropy analysis Table S2: Tokens obtained through BPE tokenization of the entire data set (PDF)
File S1. Epitope data set raw data (TXT)
File S2. Epitope tokenization raw data (TXT)

## References

[ref1] Forsström B.; Bisławska Axnäs B.; Rockberg J.; Danielsson H.; Bohlin A.; Uhlen M. Dissecting Antibodies with Regards to Linear and Conformational Epitopes. PloS One 2015, 10 (3), e0121673 10.1371/journal.pone.0121673. 25816293 PMC4376703

[ref2] Poh C. M.; Carissimo G.; Wang B.; Amrun S. N.; Lee C. Y.-P.; Chee R. S.-L.; Fong S.-W.; Yeo N. K.-W.; Lee W.-H.; Torres-Ruesta A. Two Linear Epitopes on the SARS-CoV-2 Spike Protein That Elicit Neutralising Antibodies in COVID-19 Patients. Nat. Commun. 2020, 11 (1), 1–7. 10.1038/s41467-020-16638-2. 32483236 PMC7264175

[ref3] Palatnik-de-Sousa C. B.; Soares I. d. S.; Rosa D. S. Editorial: Epitope Discovery and Synthetic Vaccine Design. Front. Immunol. 2018, 9, Article 826 10.3389/fimmu.2018.00826. PMC5915546 29720983

[ref4] Lin M. J.; Svensson-Arvelund J.; Lubitz G. S.; Marabelle A.; Melero I.; Brown B. D.; Brody J. D. Cancer Vaccines: The next Immunotherapy Frontier. Nat. Cancer 2022, 3 (8), 911–926. 10.1038/s43018-022-00418-6. 35999309

[ref5] Abbott W. M.; Damschroder M. M.; Lowe D. C. Current Approaches to Fine Mapping of Antigen–Antibody Interactions. Immunology 2014, 142 (4), 526–535. 10.1111/imm.12284. 24635566 PMC4107663

[ref6] Li N.; Li Z.; Fu Y.; Cao S. Cryo-EM Studies of Virus-Antibody Immune Complexes. Virol. Sin. 2020, 35, 1–13. 10.1007/s12250-019-00190-5. 31916022 PMC7035235

[ref7] Noren K. A.; Noren C. J. Construction of High-Complexity Combinatorial Phage Display Peptide Libraries. Methods 2001, 23 (2), 169–178. 10.1006/meth.2000.1118. 11181036

[ref8] Carter J. M. Epitope Mapping of a Protein Using the Geysen (PEPSCAN) Procedure. Protein Protoc. Handb. 1996, 581–593. 10.1007/978-1-60327-259-9_95. 7535161

[ref9] Yang K. K.; Wu Z.; Arnold F. H. Machine-Learning-Guided Directed Evolution for Protein Engineering. Nat. Methods 2019, 16 (8), 687–694. 10.1038/s41592-019-0496-6. 31308553

[ref10] Wittmann B. J.; Johnston K. E.; Wu Z.; Arnold F. H. Advances in Machine Learning for Directed Evolution. Curr. Opin. Struct. Biol. 2021, 69, 11–18. 10.1016/j.sbi.2021.01.008. 33647531

[ref11] de Jongh R. P.; van Dijk A. D.; Julsing M. K.; Schaap P. J.; de Ridder D. Designing Eukaryotic Gene Expression Regulation Using Machine Learning. Trends Biotechnol. 2020, 38 (2), 191–201. 10.1016/j.tibtech.2019.07.007. 31431299

[ref12] Moussa S.; Kilgour M.; Jans C.; Hernandez-Garcia A.; Cuperlovic-Culf M.; Bengio Y.; Simine L. Diversifying Design of Nucleic Acid Aptamers Using Unsupervised Machine Learning. J. Phys. Chem. B 2023, 127, 62–68. 10.1021/acs.jpcb.2c05660. 36574492

[ref13] Lin Z.; Akin H.; Rao R.; Hie B.; Zhu Z.; Lu W.; Smetanin N.; Verkuil R.; Kabeli O.; Shmueli Y.; dos Santos Costa A.; Fazel-Zarandi M.; Sercu T.; Candido S.; Rives A. Evolutionary-Scale Prediction of Atomic-Level Protein Structure with a Language Model. Science 2023, 379 (6637), 1123–1130. 10.1126/science.ade2574. 36927031

[ref14] Madani A.; Krause B.; Greene E. R.; Subramanian S.; Mohr B. P.; Holton J. M.; Olmos J. L.; Xiong C.; Sun Z. Z.; Socher R.; Fraser J. S.; Naik N. Large Language Models Generate Functional Protein Sequences across Diverse Families. Nat. Biotechnol. 2023, 41, 1099–1106. 10.1038/s41587-022-01618-2. 36702895 PMC10400306

[ref15] Vita R.; Mahajan S.; Overton J. A.; Dhanda S. K.; Martini S.; Cantrell J. R.; Wheeler D. K.; Sette A.; Peters B. The Immune Epitope Database (IEDB): 2018 Update. Nucleic Acids Res. 2019, 47 (D1), D339–D343. 10.1093/nar/gky1006. 30357391 PMC6324067

[ref16] Soga S.; Kuroda D.; Shirai H.; Kobori M.; Hirayama N. Use of Amino Acid Composition to Predict Epitope Residues of Individual Antibodies. Protein Eng. Des. Sel. 2010, 23 (6), 441–448. 10.1093/protein/gzq014. 20304974

[ref17] Akbar R.; Robert P. A.; Pavlović M.; Jeliazkov J. R.; Snapkov I.; Slabodkin A.; Weber C. R.; Scheffer L.; Miho E.; Haff I. H.; Haug D. T. T.; Lund-Johansen F.; Safonova Y.; Sandve G. K.; Greiff V. A Compact Vocabulary of Paratope-Epitope Interactions Enables Predictability of Antibody-Antigen Binding. Cell Rep. 2021, 34, 108856 10.1016/j.celrep.2021.108856. 33730590

[ref18] Anjana R.; Vaishnavi M. K.; Sherlin D.; Kumar S. P.; Naveen K.; Kanth P. S.; Sekar K. Aromatic-Aromatic Interactions in Structures of Proteins and Protein-DNA Complexes: A Study Based on Orientation and Distance. Bioinformation 2012, 8 (24), 1220–1224. 10.6026/97320630081220. 23275723 PMC3530875

[ref19] Arzhanik V.; Svistunova D.; Koliasnikov O.; Egorov A. M. Interaction of Antibodies with Aromatic Ligands: The Role of the π-Stacking. J. Bioinform Comput. Biol. 2010, 08 (03), 471–483. 10.1142/S0219720010004835. 20556857

[ref20] Meyer E. A.; Castellano R. K.; Diederich F. Interactions with Aromatic Rings in Chemical and Biological Recognition. Angew. Chem., Int. Ed. Engl. 2003, 42 (11), 1210–1250. 10.1002/anie.200390319. 12645054

[ref21] Lanzarotti E.; Defelipe L. A.; Marti M. A.; Turjanski A. G. Aromatic Clusters in Protein-Protein and Protein-Drug Complexes. J. Cheminformatics 2020, 12 (1), Article 30 10.1186/s13321-020-00437-4. PMC7206889 33431014

[ref22] Cremlyn R. J.; Cremlyn R. J. W. An Introduction to Organosulfur Chemistry; John Wiley & Sons, 1996.

[ref23] Magliery T. J.; Regan L. Sequence Variation in Ligand Binding Sites in Proteins. BMC Bioinformatics 2005, 6 (1), Article 240 10.1186/1471-2105-6-240. 16194281 PMC1261162

[ref24] Madeira F.; Park Y. m.; Lee J.; Buso N.; Gur T.; Madhusoodanan N.; Basutkar P.; Tivey A. R N; Potter S. C; Finn R. D; Lopez R. The EMBL-EBI Search and Sequence Analysis Tools APIs in 2019. Nucleic Acids Res. 2019, 47 (W1), W636–W641. 10.1093/nar/gkz268. 30976793 PMC6602479

[ref25] Mayrose I.; Shlomi T.; Rubinstein N. D.; Gershoni J. M.; Ruppin E.; Sharan R.; Pupko T. Epitope Mapping Using Combinatorial Phage-Display Libraries: A Graph-Based Algorithm. Nucleic Acids Res. 2007, 35 (1), 69–78. 10.1093/nar/gkl975. 17151070 PMC1761437

[ref26] Chen W. H.; Sun P. P.; Lu Y.; Guo W. W.; Huang Y. X.; Ma Z. Q. MimoPro: A More Efficient Web-Based Tool for Epitope Prediction Using Phage Display Libraries. BMC Bioinformatics 2011, 12 (1), 199 10.1186/1471-2105-12-199. 21609501 PMC3124435

[ref27] Inza I.; Calvo B.; Armañanzas R.; Bengoetxea E.; Larranaga P.; Lozano J. A. Machine Learning: An Indispensable Tool in Bioinformatics. Bioinformatics methods in clinical research; Springer, 2010; pp 25–48. 10.1007/978-1-60327-194-3_2 19957143

[ref28] Ferruz N.; Höcker B. Controllable Protein Design with Language Models. Nat. Mach. Intell. 2022, 4 (6), 521–532. 10.1038/s42256-022-00499-z.

[ref29] Ofer D.; Brandes N.; Linial M. The Language of Proteins: NLP, Machine Learning & Protein Sequences. Comput. Struct. Biotechnol. J. 2021, 19, 1750–1758. 10.1016/j.csbj.2021.03.022. 33897979 PMC8050421

[ref30] Lin Z.; Akin H.; Rao R.; Hie B.; Zhu Z.; Lu W.; dos Santos Costa A.; Fazel-Zarandi M.; Sercu T.; Candido S. Language Models of Protein Sequences at the Scale of Evolution Enable Accurate Structure Prediction. bioRxiv, July 21, 2022. 10.1101/2022.07.20.500902

[ref31] Gage P. A New Algorithm for Data Compression. The C Users J. 1994, 12 (2), 23–38.

[ref32] Hughes J.; Piltz S.; Rogers N.; McAninch D.; Rowley L.; Thomas P. Mechanistic Insight into the Pathology of Polyalanine Expansion Disorders Revealed by a Mouse Model for X Linked Hypopituitarism. PLOS Genet. 2013, 9 (3), e1003290 10.1371/journal.pgen.1003290. 23505376 PMC3591313

[ref33] Liufu T.; Zheng Y.; Yu J.; Yuan Y.; Wang Z.; Deng J.; Hong D. The PolyG Diseases: A New Disease Entity. Acta Neuropathol. Commun. 2022, 10 (1), 79 10.1186/s40478-022-01383-y. 35642014 PMC9153130

[ref34] Bartholomeu D. C.; Cerqueira G. C.; Leão A. C. A.; daRocha W. D.; Pais F. S.; Macedo C.; Djikeng A.; Teixeira S. M. R.; El-Sayed N. M. Genomic Organization and Expression Profile of the Mucin-Associated Surface Protein (Masp) Family of the Human Pathogen Trypanosoma Cruzi. Nucleic Acids Res. 2009, 37 (10), 3407–3417. 10.1093/nar/gkp172. 19336417 PMC2691823

[ref35] Nakashima H.; Ota M.; Nishikawa K.; Ooi T. Genes from Nine Genomes Are Separated into Their Organisms in the Dinucleotide Composition Space. DNA Res. 1998, 5 (5), 251–259. 10.1093/dnares/5.5.251. 9872449

[ref36] Piontkivska H.; Hughes A. L. Patterns of Sequence Evolution at Epitopes for Host Antibodies and Cytotoxic T-Lymphocytes in Human Immunodeficiency Virus Type 1. Virus Res. 2006, 116 (1), 98–105. 10.1016/j.virusres.2005.09.001. 16214253

[ref37] Thörnqvist L.; Sjöberg R.; Greiff L.; van Hage M.; Ohlin M. Linear Epitope Binding Patterns of Grass Pollen-Specific Antibodies in Allergy and in Response to Allergen-Specific Immunotherapy. Front. Allergy 2022, 3, Article 859126 10.3389/falgy.2022.859126. 35769580 PMC9234942

[ref38] Constant D. A.; Gutierrez J. M.; Sastry A. V.; Viazzo R.; Smith N. R.; Hossain J.; Spencer D. A.; Carter H.; Ventura A. B.; Louie M. T. Deep Learning-Based Codon Optimization with Large-Scale Synonymous Variant Datasets Enables Generalized Tunable Protein Expression. bioRxiv, Feb. 12, 2023. 10.1101/2023.02.11.528149.

[ref39] Peng H.-P.; Lee K. H.; Jian J.-W.; Yang A.-S. Origins of Specificity and Affinity in Antibody–Protein Interactions. Proc. Natl. Acad. Sci. U. S. A. 2014, 111 (26), E2656–E2665. 10.1073/pnas.1401131111. 24938786 PMC4084487

[ref40] Traxlmayr M. W.; Kiefer J. D.; Srinivas R. R.; Lobner E.; Tisdale A. W.; Mehta N. K.; Yang N. J.; Tidor B.; Wittrup K. D. Strong Enrichment of Aromatic Residues in Binding Sites from a Charge-Neutralized Hyperthermostable Sso7d Scaffold Library. J. Biol. Chem. 2016, 291 (43), 22496–22508. 10.1074/jbc.M116.741314. 27582495 PMC5077188

[ref41] Zavrtanik U.; Lukan J.; Loris R.; Lah J.; Hadži S. Structural Basis of Epitope Recognition by Heavy-Chain Camelid Antibodies. J. Mol. Biol. 2018, 430 (21), 4369–4386. 10.1016/j.jmb.2018.09.002. 30205092

[ref42] Magliery T. J.; Regan L. Beyond Consensus: Statistical Free Energies Reveal Hidden Interactions in the Design of a TPR Motif. J. Mol. Biol. 2004, 343 (3), 731–745. 10.1016/j.jmb.2004.08.026. 15465058

[ref43] Tsuji J.; Nydza R.; Wolcott E.; Mannor E.; Moran B.; Hesson G.; Arvidson T.; Howe K.; Hayes R.; Ramirez M.; Way M. The Frequencies of Amino Acids Encoded by Genomes That Utilize Standard and Nonstandard Genetic Codes. Bios 2010, 81 (1), 22–31. 10.1893/011.081.0103.

[ref44] Wolf T.; Debut L.; Sanh V.; Chaumond J.; Delangue C.; Moi A.; Cistac P.; Rault T.; Louf R.; Funtowicz M. Transformers: State-of-the-Art Natural Language Processing. Proceedings of the 2020 Conference on Empirical Methods in Natural Language Processing: System Demonstrations; Association for Computational Linguistics, 2020; 38–45. 10.18653/v1/2020.emnlp-demos.6.

