# Supplementary material for: Statistical Analysis and Tokenization of Epitopes to Construct Artificial Neoepitope Libraries
Source: ACS Synth Biol. 2023 Sep 13;12(10):2812–8. doi: 10.1021/acssynbio.3c00201 (PMC10594869; doi:10.1021/acssynbio.3c00201)
Supplement: Supplementary file 1 — sb3c00201_si_001.pdf [file sb3c00201_si_001.pdf]

Supporting information for:

Statistical Analysis and Tokenization of Epitopes to Construct Artificial Neoepitope Libraries

Elena Lopez-Martinez,[a‡] Aitor Manteca,[a‡] Noelia Ferruz,[b,*] and Aitziber L. Cortajarena[a,c,*]

[a] Centre for Cooperative Research in Biomaterials (CIC biomaGUNE), Basque Research and Technology Alliance (BRTA), Paseo de Miramón 194, Donostia-San Sebastián, 20014 Spain;

[b] Institut de Biologia Molecular de Barcelona IBMB, Baldiri Reixac 4-8, Tower R, 3rd Floor, Barcelona, Spain;

[c] IKERBASQUE, Basque Foundation for Science. Plaza Euskadi 5, 48009 Bilbao, Spain.

*Corresponding authors (emails: noelia.ferruz@ibmb.csic.es and alcortajarena@cicbiomagune.es)

Contents:

1. Figure S1. Amino acid global propensity in epitopes. S2
2. Figure S2. Overall global propensity variation in epitopes. S3
3. Figure S3. Relative entropy values for the epitopes. S4
4. Figure S4. Distribution of epitopes by source organism. S5
5. Figure S5. Tokenization of epitopes with tokens of 4 and 5 residues. S6
6. Figure S6. Amino acid frequency after tokenization. S7
7. Table S1: Average relative entropies and p-values for the entropy analysis. S8
8. Table S2: Tokens obtained through BPE tokenization of the entire dataset. S9

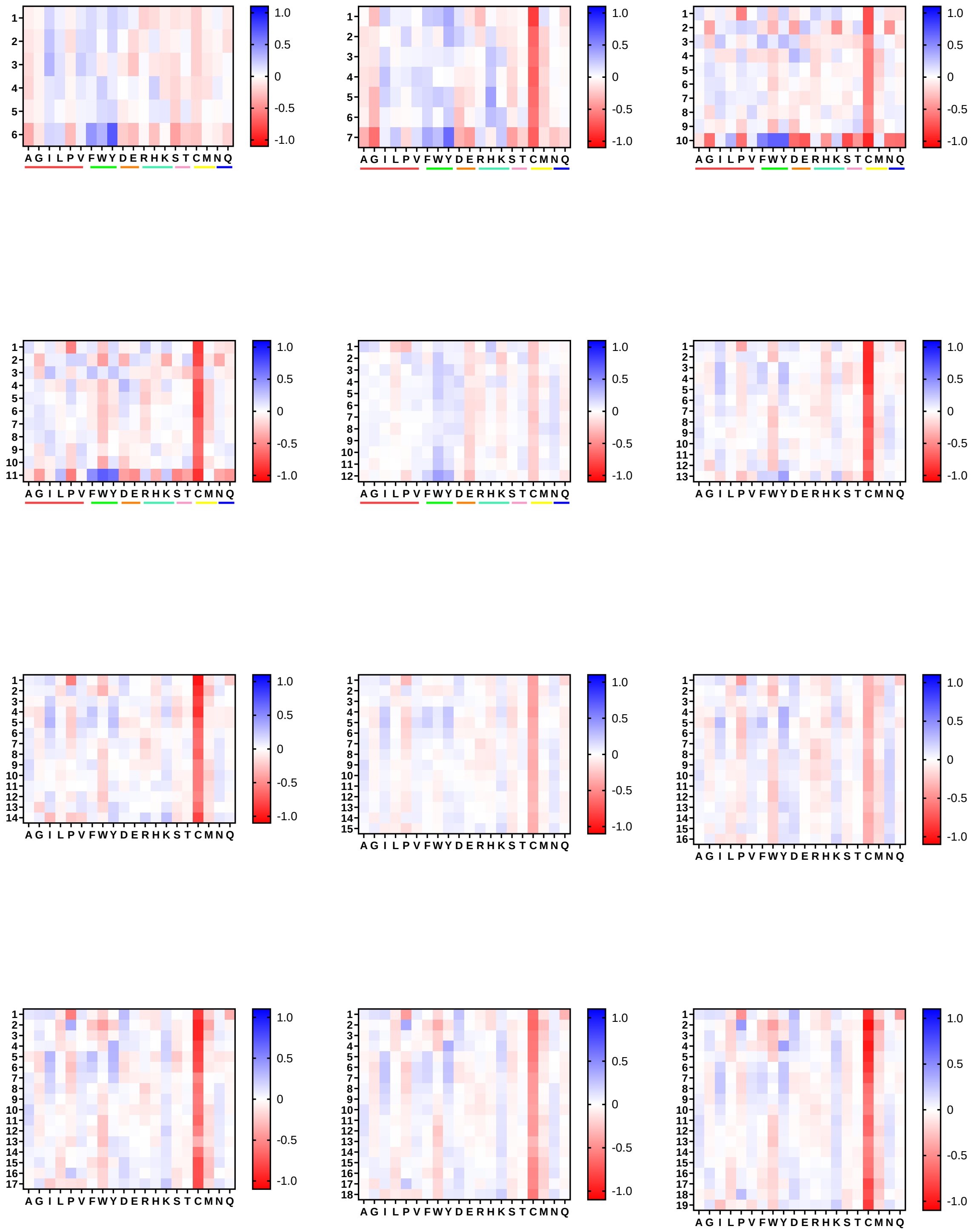

Figure S1. Amino acid global propensity in epitopes. The propensity of each amino acid at each position is shown from 6-mer to 19-mer epitopes, excluding 8-mers and 9-mers.

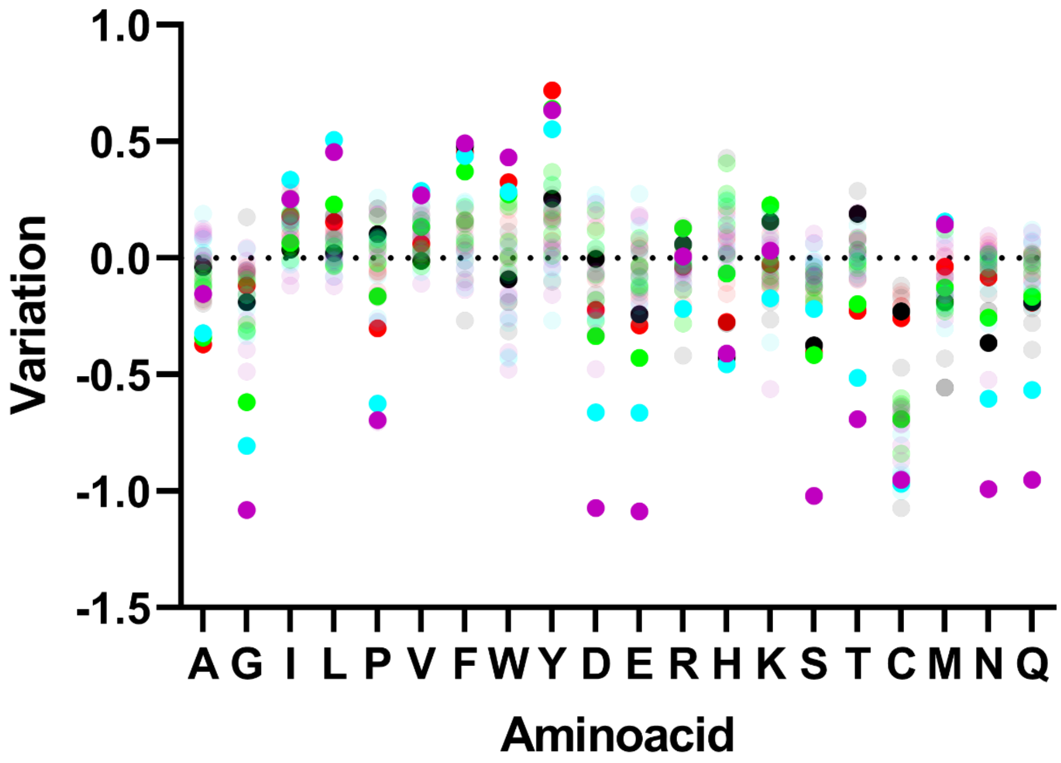

Figure S2. Overall global propensity variation in epitopes. Each dot represents an x-mer that shows its global propensity for all the amino acids. The increase in the propensity of aromatic residues and the decrease of cysteines can be observed.

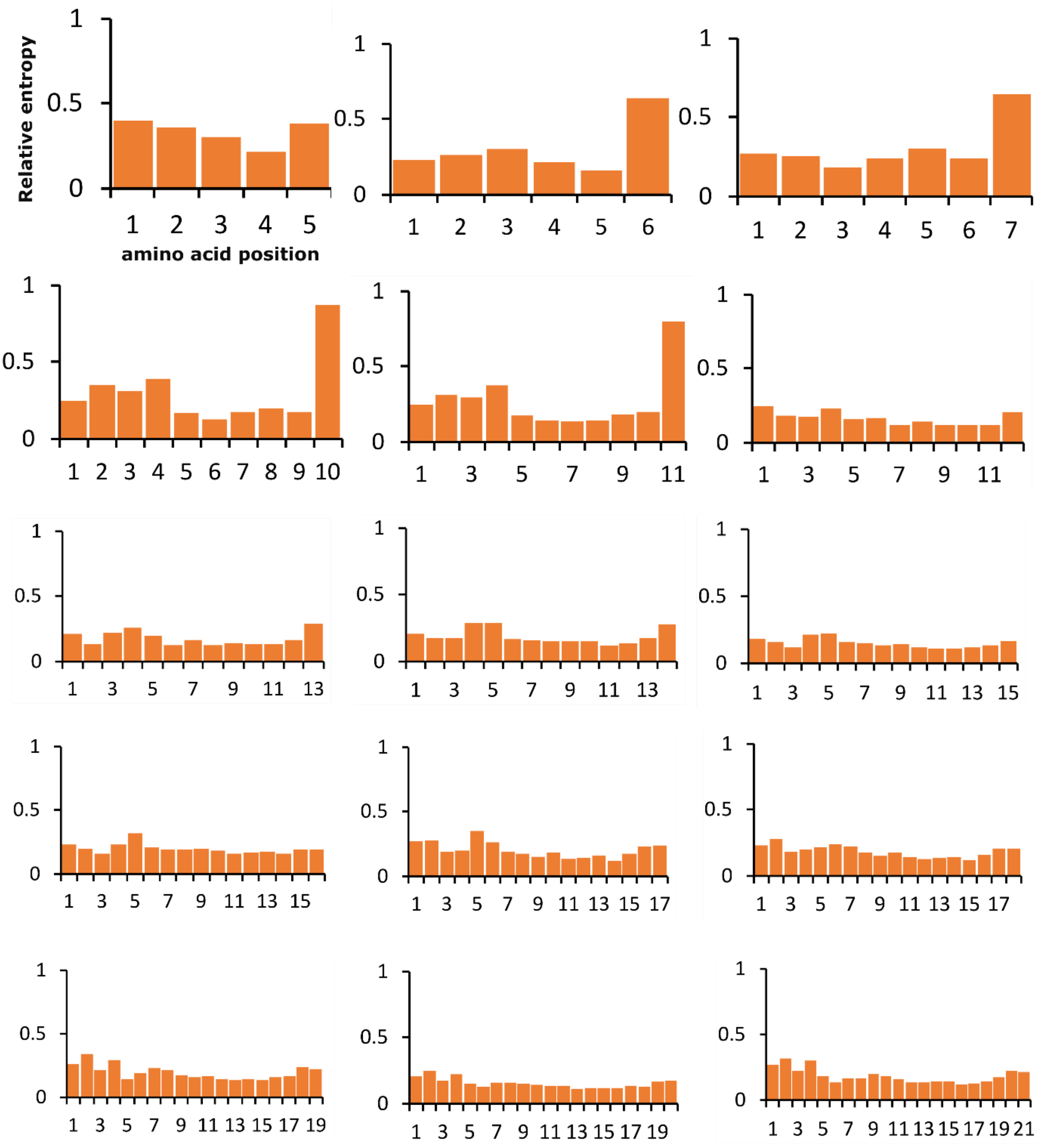

Figure S3. Relative entropy values for the epitopes. The entropy is shown for each position of the epitope is shown from 6-mer to 21-mer epitopes, excluding 8-mers and 9-mers.

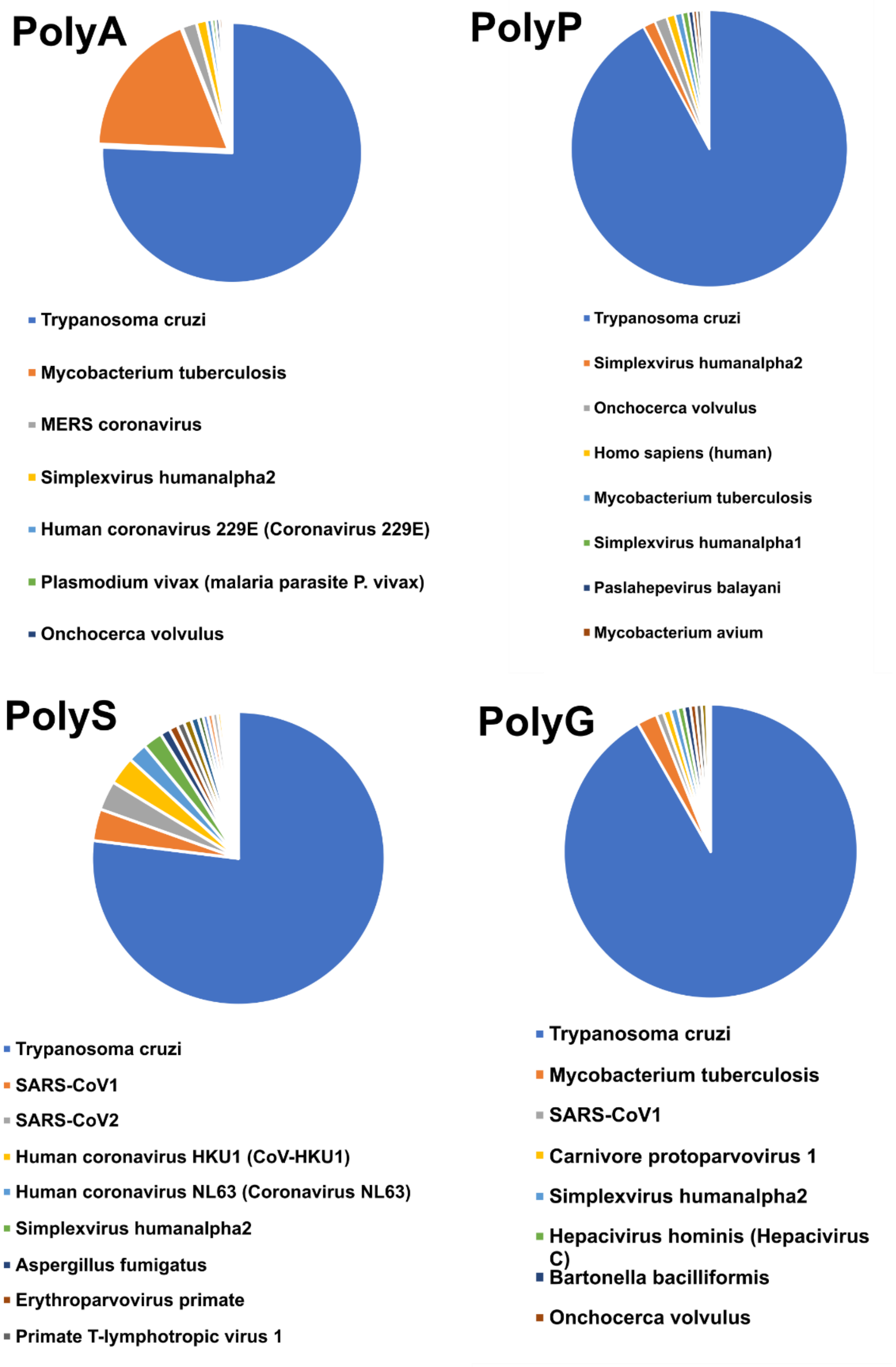

Figure S4. Distribution of epitopes by source organism. The epitopes containing a 4mer polyX in their sequences for polyA, polyP, polyS, and polyG are shown in their corresponding pie charts. At least 75% of the sequences correspond to *Trypanosoma cruzi* for all the polyXs.

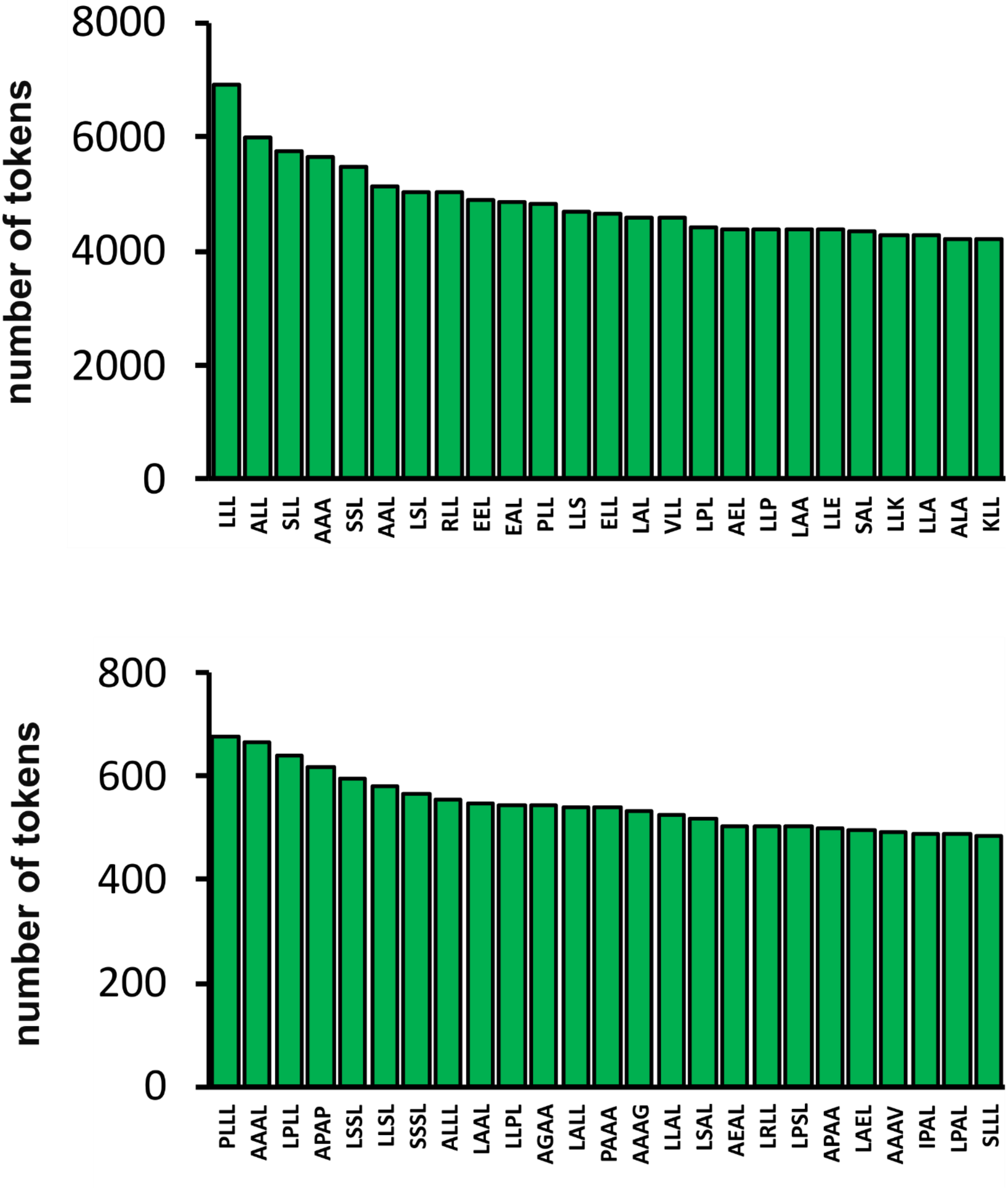

Figure S5. Tokenization of epitopes with tokens of 3 and 4 residues. The 25 most represented tokens with sizes of 3 and 4 residues. It is important to note that the presence of polyalanine, polyproline, and polyleucine segments in the data introduces noise to the analysis and avoids the identification of representative epitope segments.

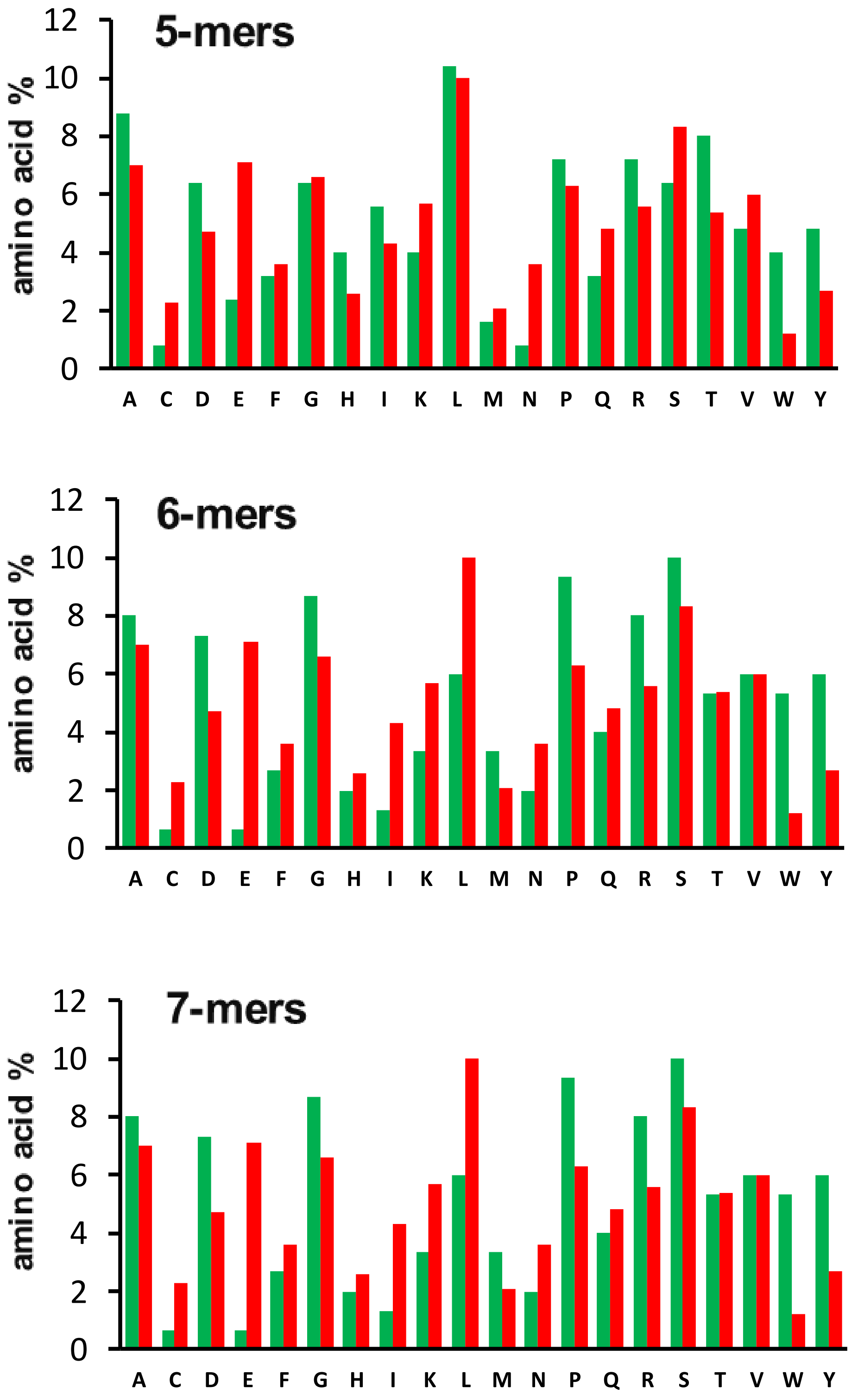

Figure S6. Amino acid frequency after tokenization. The frequency of the 25 most represented tokens is shown in green whereas the natural frequency is depicted in red.

Table S1. Average relative entropies and p-values for the entropy analysis. The p-values of relative entropy are obtained with a single factor ANOVA analysis using a significance level α = 0.05. The pale green values correspond to p-values < 0.05, and the bright green values correspond to p-values < 0.01.

nºaa	sequences	Average Rel. Ent.	p-values
5	514	0.33	0.575807458
6	1809	0.30	0.027375324
7	6607	0.31	**0.003525015**
8	55609	0.36	**0.003229473**
9	268118	0.31	**0.000663313**
10	104874	0.30	**1.5215E-05**
11	68485	0.27	**3.33749E-07**
12	120790	0.17	0.094951322
13	47663	0.18	0.02943571
14	48526	0.19	0.034806227
15	90395	0.15	0.137247936
16	70417	0.20	0.354467941
17	36808	0.20	0.165530786
18	30463	0.18	0.60054662
19	18073	0.20	0.592997674
20	20250	0.15	0.6562244
21	10219	0.19	0.396400169

Table S2. Tokens obtained through BPE tokenization of the entire dataset. Tokens are classified by their length (2 to 10 amino acids-long) for different vocabulary sizes. The first column, including tokens of a single residue has a constant value of 29 (including special amino acids and characters) and is not included for clarity. Coverage_1, Coverage_2 and Coverage_3 show the dataset coverage (%) for tokens of the largest, second largest and third largest sizes on each row.

	**di-**	**tri-**	**tetra-**	**penta-**	**hexa-**	**hepta-**	**octo-**	**nona-**	**deca-**	**coverage_1 (%)**	**coverage_2 (%)**	**coverage_3 (%)**
**50**	21	0	0	0	0	0	0	0	0	75.01	100.0	100.0
**1000**	356	615	0	0	0	0	0	0	0	83.64	100.0	100.0
**1500**	369	1100	2	0	0	0	0	0	0	0.14	94.78	100.0
**2000**	379	1567	25	0	0	0	0	0	0	1.4	98.28	100.0
**3000**	385	2385	192	7	0	2	0	0	0	0.05	0.05	0.19
**4000**	386	2985	570	18	6	5	1	0	0	0.02	0.11	0.2
**5000**	388	3435	1098	27	13	8	2	0	0	0.03	0.16	0.33
**6000**	388	3808	1699	41	21	10	4	0	0	0.05	0.2	0.44
**7000**	390	4117	2348	63	28	17	6	1	1	0.01	0.01	0.08
**8000**	390	4363	3073	75	35	23	8	3	1	0.01	0.03	0.11
**9000**	391	4594	3792	100	43	27	13	9	2	0.01	0.07	0.18
**9999**	391	4771	4543	134	56	41	21	11	2	0.01	0.09	0.24
